# Complete blood count data and leukocyte expression of cytokine genes and cytokine receptor genes associated with bovine respiratory disease in calves

**DOI:** 10.1186/s13104-018-3900-x

**Published:** 2018-11-03

**Authors:** Amanda K. Lindholm-Perry, Larry A. Kuehn, Tara G. McDaneld, Jeremy R. Miles, Aspen M. Workman, Carol G. Chitko-McKown, John W. Keele

**Affiliations:** USDA, ARS, U.S. Meat Animal Research Center, P.O. Box 166, Clay Center, NE 68933 USA

**Keywords:** Beef cattle, BRD, Gene expression, Pre-conditioning, qRT-PCR, WBC, Weaning, Leukocyte, CBC

## Abstract

**Objective:**

The purpose of this study was to evaluate potential relationships between cytokine gene expression, complete blood counts (CBC) and animals that were sick or would become sick. The CBC and the transcript abundance of cytokines and their receptors expressed in leukocytes were measured from calves at two early timepoints, and again after diagnosis with bovine respiratory disease (BRD).

**Results:**

Blood was collected from calves at pre-conditioning (n = 796) and weaning (n = 791) for CBC. Blood counts were also measured for the calves with BRD (n = 13), and asymptomatic calves (n = 75) after weaning. The CBC were compared for these animals at 3 time points. At diagnosis, neutrophils were higher and basophils lower in sick animals (P < 0.05). To further characterize BRD responses, transcript abundance of 84 cytokine genes were evaluated in 5 calves with BRD and 9 asymptomatic animals at all time points. There was more data for CBC than transcript abundance; hence, animal and temporary environmental correlations between CBC and transcript abundance were exploited to improve the power of the transcript abundance data. Expression of *CCL16*, *CXCR1*, *CCR1* was increased in BRD positive animals compared to controls (P-corrected < 0.1). Cytokine expression data may help to provide insight into an animal’s health.

**Electronic supplementary material:**

The online version of this article (10.1186/s13104-018-3900-x) contains supplementary material, which is available to authorized users.

## Introduction

The ability to predict whether an animal is susceptible to bovine respiratory disease (BRD) would benefit producers. Cost savings may be realized by managing animals with higher potential susceptibility to BRD differently by attempting to reduce their exposure to illness or by monitoring susceptible animals more closely for illness. Alternatively, these animals might be treated prophylactically at an earlier stage to mitigate the risk of illness.

Previously, Leach et al. [1] described relationships between changes in WBC and lymphocytes in response to vaccine and health records in calves. White blood cells use small pro- and anti-inflammatory cytokines as molecular signaling mechanisms to communicate with each other. Cytokines become elevated in response to the bacterial and viral causes of BRD [2–4]. The profiles of the most studied cytokines (TNF, INFγ, IL-1β, IL-6) vary depending on which pathogen the animal is infected with [4]. The purpose of this study was to evaluate the transcript abundance of multiple cytokine genes and receptors in calves at different production stages (i.e. preconditioning and weaning), and when BRD was detected to determine whether changes in blood cell counts and/or changes in the expression of specific cytokines might indicate which animals were sick or more likely to become sick with BRD.

## Main text

### Population

Heifer and steer calves for this study were born in the fall of 2016 and were from the continuous phase of the USMARC Germplasm Evaluation project [5]. This breeding population includes contributions from the following 18 breeds: Angus, Beefmaster, Brahman, Brangus, Braunvieh, Charolais, Chiangus, Gelbvieh, Hereford, Limousin, Maine Anjou, Red Angus, Salers, Santa Gertrudis, Shorthorn, South Devon, Simmental, and Tarentaise. Calves were part of a subset developed to monitor disease. Calves were all born and raised at USMARC at three locations with no fenceline contact. At pre-conditioning (~ 120 days of age), which is preweaning vaccination with Bovi Shield Gold One Shot (Zoetis) and Vision 8 with SPUR (Merck, USA) and at weaning (~ 150 days) with the vaccination Bovi Shield Gold One Shot, blood was collected as previously described [1]. Briefly, whole blood was collected into tubes containing liquid EDTA as an anticoagulant for a complete blood count (CBC) from 796 animals at pre-conditioning and 791 animals at weaning, when calves were relocated from pasture to a feedlot. At the same times, whole blood from a randomly selected subset of these animals (n = 200) was also collected into Tempus RNA Blood tubes (Thermo, Waltham, MA, USA) for RNA isolation. Samples in Tempus tubes were stored at − 20 °C prior to RNA isolation. After weaning, calves were monitored for 42 days for clinical signs of BRD, including cough, nasal discharge, increased respiratory rate, and lethargy. Whole blood from calves diagnosed with BRD (n = 13) and asymptomatic control animals (n = 75) were collected as described above for CBC. If the animal with BRD was previously included in the subset of animals with blood drawn into Tempus tubes, another Tempus tube was drawn at the time of diagnosis (n = 5). Healthy, control animals from the same or nearby pens that had samples previously collected in Tempus tubes (n = 9), were also collected into a new Tempus blood tube. Nasal swabs were also collected from the upper nasal cavity of case and control animals for pathogen detection.

### Hematology analysis at pre-conditioning and weaning

All freshly collected whole blood samples with EDTA were evaluated using a HT5 veterinary hematology instrument (Heska, Loveland, CO, USA) and cell component data was recorded. The average, minimum and maximum values for the whole blood parameters are reported for all animals at pre-conditioning (n = 796) and at weaning (n = 791) in Table 1. Most of the average values obtained fell within the reference intervals of at least one of three bovine studies presented in Roland et al. [6] and Leach et al. [1]. All values fell within the reference intervals provided by hematologic reference chart in the Merck Veterinary Manual. Differences between this study and those described in Roland et al. [6] included slightly higher values for erythrocytes (10.9 × 10^12^/L at pre-conditioning and 10.6 × 10^12^/L at weaning), higher red cell distribution width (24.8% at pre-conditioning and 24.9% at weaning), and lower average values for mean corpuscular volume (34.7 fL at pre-conditioning, 35.1 fL at weaning). The CBC values for pre-conditioning and weaning were within the ranges of those presented in [1], except for eosinophils, which were lower in the animals presented in this study.

**Table 1:** Averages for the whole blood parameters for crossbred beef calves at preconditioning (n = 796; approximately 120 days) and at weaning (n = 790; 150 days)

Parameter	Preconditioning			Weaning		
	Average (SD)	Minimum	Maximum	Average (SD)	Minimum	Maximum
WBC (10^9^/L)	11.1 (2.5)	4.71	22.66	11.3 (2.5)	4.98	23.7
NEU (10^9^/L)	3.3 (1.4)	0.74	10.82	3.5 (1.4)	0.85	15.33
LYM (10^9^/L)	7.0 (1.7)	2.85	17.63	7.0 (1.9)	2.44	18.87
MONO (10^9^/L)	0.55 (0.2)	0.11	1.51	0.59 (0.21)	0.17	1.7
EOS (10^9^/L)	0.15 (0.11)	0.02	1.18	0.14 (0.074)	0.03	1.02
BAS (10^9^/L)	0.09 (0.04)	0	0.32	0.096 (0.043)	0.02	0.38
NEU%	29.4 (8.1)	8.2	60.2	30.5 (8.6)	9.3	64.9
LYM%	63.5 (8.6)	33.2	88.5	62.2 (8.99)	30.6	85.3
MONO%	4.9 (1.5)	1.3	11.8	5.3 (1.7)	1.8	12.7
EOS%	1.4 (0.9)	0.4	10.9	1.27 (0.64)	0.3	8.7
BAS%	0.8 (0.3)	0	2.4	0.85 (0.35)	0.2	2.6
RBC (10^12^/L)	10.9 (1.0)	3.7	14.57	10.6 (1.1)	7.38	14
HGB (g/dL)	12.96 (0.9)	5.1	15.7	12.5 (0.9)	9.4	15.5
HCT%	37.8 (2.7)	14	46.4	37.0 (2.6)	27.6	45.6
MCV (fL)	34.7 (2.4)	28	43.1	35.1 (2.5)	27.9	45.6
MCH (pg)	11.9 (0.8)	9.7	15	11.8 (0.9)	9.3	15.1
MCHC (g/dL)	34.3 (0.9)	31.2	36.6	33.6 (0.96)	31	37.8
RDW%	24.8 (1.9)	21.1	35.4	24.9 (1.8)	20.7	33.8
PLT (10^9^/L)	721.7 (244.9)	34	1692	592.0 (182.8)	24	1450
MPV (fL)	4.7 (0.3)	3.8	5.7	4.7 (0.3)	3.9	5.8

*WBC* white blood cells, *NEU* neutrophils, *LYM* lymphocytes, *MONO* monocytes, *EOS* eosinophils, *BAS* basophils, *RBC* red blood cells, *HGB* hemoglobin, *HCT* hematocrit, *MCV* mean corpuscular volume, *MCH* mean corpuscular hemoglobin, *MCHC* mean corpuscular hemoglobin concentration, *RDW* red cell distribution width, *PLT* platelets, *MPV* mean platelet volume

### Cytokine gene expression

Total RNA was isolated from the whole blood collected in Tempus tubes drawn at the three time points (preconditioning, weaning and diagnosis) from the animals diagnosed with disease (n = 5) and control animals (n = 9). The Tempus Spin RNA isolation kit (Thermo) was used to isolate RNA following the manufacturer’s instructions. Quality of RNA was assessed using a Bioanalyzer 2200 Tape Station (Agilent, Santa Clara, CA, USA). All samples produced RNA integrity values (RIN) of > 8. The RT^2^ First Strand Kit (Qiagen, Germantown, MD) was used for cDNA preparation from 1 μg of total RNA. The cDNA was diluted with water and added to the RT^2^ SYBR Green Mastermix for a final reaction volume of 25 μL. The master mix with cDNA was placed into the wells of the Bovine Inflammatory Cytokine and Receptor PCR Array (Catalog #PABT-011Z; Qiagen) plates for the purpose of assessing the level of transcript abundance of 84 different immune genes. Thermal cycling conditions on a CFX96 Touch Real-Time PCR Detection System (Bio-Rad, Hercules, CA, USA) were: 95 °C for 10 min followed by 40 cycles of 95 °C for 15 s and 60 °C for 1 min. Data were entered into the Qiagen RT2 profiler data analysis software (http://pcrdataanalysis.sabiosciences.coms/pcr/arrayanalysis.php). The gene *hypoxanthine phosphoribosyltransferase 1* (*HPRT1*) was chosen as the housekeeping gene for normalization because it was the most stable of the five housekeeping genes on the plates between the BRD positive and control animals. The 2^−ΔΔCT^ method [7] was used to calculate the expression values for all 84 genes and these values were used to evaluate these genes for differential expression between sick and asymptomatic animals.

### Statistical analysis of hematology and cytokine gene expression

Multiple trait mixed model analyses were applied to hematological and cytokine (both ligand and receptor) transcript abundance. Each cytokine was analyzed in a separate mixed model analysis with 5 hematological cell counts: neutrophils, lymphocytes, monocytes, eosinophils, and basophils. Six cytokines or receptors were eliminated from mixed model analysis because of more than 5 missing values (> 10% missing); hence, we ran 78 mixed model analyses. For each analysis we included 36 equations for fixed effects; 4 phenotype (sick verses healthy) by time point subclasses times 6 traits; 5 CBC cell counts and transcript abundance for one cytokine. For random animal effects there were 534 equations, 89 animals times 6 traits. In a 6 × 6 variance covariance matrix, there are 21 unique variances and covariances. We estimated 2 variance–covariance matrices, one for animal effects and 1 for temporary environmental effects for a total of 42 variances and covariances. We used REML combined with a Cholesky decomposition to constrain the variance matrix to be positive definite in R using optim() using method = “L-BFGS-B. Differences between sick and healthy animals at diagnosis were tested using an F test with 1 *df* for the numerator and 83 *df* for the denominator for cell counts and 8 *df* for the denominator for cytokine expression. Experiment-wise type I error was controlled for multiple testing of 84 genes using a Bonferroni adjustment. The multiple trait mixed model analysis incorporates indirect information from CBC to help estimate and interpret cytokine expression levels where information was more limited.

### Hematology analysis in BRD case vs. control animals

Complete blood count information for sick and control animals are presented in Table 2. Lower levels of monocytes were identified at weaning (P_corrected_ ≤ 0.02) in those calves that went on to develop BRD in the feedlot compared to calves that remained healthy/asymptomatic in the first 42 days after weaning. Higher levels of neutrophils and lower levels of basophils were detected in the sick animals compared to the asymptomatic controls at the time of diagnosis (P_corrected_ ≤ 0.006). Higher neutrophil levels are indicative of inflammation related to infection caused by bacterial infection [8].

**Table 2:** Averages (SD) for the complete blood count for crossbred beef calves for diagnosed with BRD and for asymptomatic, healthy controls at each time point

Parameter	Asymptomatic control animals (n = 71)			Animals with BRD (n = 16)		
	Time 1 average (SD)^a^	Time 2	Time 3	Time 1	Time 2	Time 3
WBC	11.3 (0.29)	11.2 (0.32)	10.2 (0.29)	11.5 (0.59)	11.4 (0.65)	11.5 (0.58)
NEU	3.7 (0.14)	3.6 (0.17)	2.8 (0.17)	3.4 (0.29)	3.7 (0.35)	4.9 (0.35)
LYM	6.8 (0.21)	6.8 (0.25)	6.7 (0.21)	7.2 (0.42)	7 (0.5)	5.9 (0.43)
MONO	0.57 (0.02)	0.61 (0.022)	0.42 (0.019)	0.57 (0.04)	0.47 (0.044)	0.46 (0.038)
EOS	0.19 (0.013)	0.16 (0.007)	0.18 (0.018)	0.16 (0.027)	0.14 (0.015)	0.14 (0.036)
BAS	0.08 (0.005)	0.1 (0.004)	0.1 (0.005)	0.09 (0.01)	0.09 (0.009)	0.07 (0.011)
NEU%	32.1 (0.87)	32.1 (1.13)	27.4 (1.11)	30.2 (1.78)	31.5 (2.3)	40.8 (2.26)
LYM%	60.4 (0.88)	60.1 (1.17)	65.6 (1.1)	62.7 (1.78)	62.2 (0.38)	53 (2.23)
MONO%	5.1 (0.16)	5.5 (0.18)	4.2 (0.2)	4.9 (0.32)	4.2 (0.36)	4.3 (0.4)
EOS%	1.7 (0.11)	1.4 (0.06)	1.8 (0.16)	1.4 (0.23)	1.3 (0.12)	1.3 (0.33)
BAS%	0.73 (0.04)	0.87 (0.04)	0.99 (0.049)	0.79 (0.081)	0.77 (0.081)	0.64 (0.101)
RBC	11 (0.15)	10.5 (0.11)	11.3 (0.13)	10.9 (0.3)	10.5 (0.23)	10.8 (0.26)
HGB	13.1 (0.15)	12.5 (0.1)	13.2 (0.12)	12.9 (0.3)	12.4 (0.2)	12.6 (0.24)
HCT	38.3 (0.44)	37 (0.3)	40.4 (0.38)	37.3 (0.89)	36.4 (0.62)	37.8 (0.78)
MCV	35 (0.28)	35.4 (0.28)	35.9 (0.32)	34.5 (0.56)	34.8 (0.56)	35.3 (0.63)
MCH	12 (0.1)	11.9 (0.1)	11.8 (0.1)	11.9 (0.2)	11.9 (0.21)	11.8 (0.21)
MCHC	34.3 (0.13)	33.7 (0.11)	32.8 (0.12)	34.5 (0.26)	34 (0.22)	33.4 (0.25)
RDW%	24.8 (0.22)	25.2 (0.21)	25.4 (0.23)	25.6 (0.45)	25.4 (0.43)	25.2 (0.47)
PLT	639 (28.01)	548.8 (20.13)	676.9 (25.8)	816.6 (57.18)	630.9 (41.1)	624.6 (52.63)
MPV	4.7 (0.03)	4.8 (0.03)	4.6 (0.04)	4.7 (0.07)	4.7 (0.07)	4.5 (0.07)

*WBC* white blood cell count (10^9^/L), *NEU* neutrophil count (10^9^/L), *LYM* lymphocyte count (10^9^/L), *MONO* monocyte count (10^9^/L), *EOS* eosinophil count (10^9^/L), *BAS* basophil count (10^9^/L), *NEU%* neutrophil percent, *LYM%* lymphocyte percent, *MONO%* monocyte percent, *EOS%* eosinophil percent, *BAS%* basophil percent, *RBC* red blood cell count (10^12^/L), *PLT* platelet count (10^9^/L), *HGB* hemoglobin, *HCT* hematocrit, *MCV* mean corpuscular volume, *MCH* mean corpuscular hemoglobin, *MCHC* mean corpuscular hemoglobin concentration, *RDW* red cell distribution width, *PLT* platelets, *MPV* mean platelet volume

^a^Time 1: Pre-conditioning; Time 2: Weaning; Time 3: Diagnosis of disease and case control. Parameters are presented as average values for all animals and standard deviation in parentheses

### Pathogen detection in BRD case animals

BRD is a multi-factorial disease that can be initiated by various viruses and bacterial pathogens. Thus, diagnostic testing was performed to identify which pathogens were associated with BRD diagnosis. Multiplex reverse transcription real-time polymerase chain reaction (RT-qPCR) was used to detect bovine coronavirus (BCV), bovine respiratory syncytial virus (BRSV), bovine viral diarrhea virus (BVDV), and bovine herpesvirus-1 (BHV-1) in nasal swab samples as previously described [9]. For bacterial diagnostics, two techniques were used. First, a set of nasal swabs was sent to the University of Nebraska Lincoln Veterinary Diagnostic Center for bacterial culture and identification. Second, variable regions 1 through 3 in the 16S ribosomal RNA gene were sequenced and bacterial taxa were identified as previously described [10]. Viral pathogens associated with BRD were detected in nasal swabs from 11 of 13 cases. Six were positive for BCV, three were positive for BCV and BRSV, and two were positive for BRSV. Bacterial culture techniques identified *Pasteurella multocida*, *Mannheimia haemolytica*, or *Histophilus somni* in calves diagnosed with BRD. In addition, evaluation of the microbiome through sequencing identified predominant bacteria genera previously reported to be associated with cattle diagnosed with BRDC including *Mycoplasma* sp. and *Psychrobacter* sp. [10].

### Leukocyte gene expression analysis from sick and case control animals

Three genes were identified in this study as differentially expressed between sick and healthy control animals at the time of diagnosis of disease. These genes included *CCL16*, *CXCR1*, and *CCR1* (P_corrected_ < 0.1; Table 3). This is the first study to present a potential association between the expression of these genes with BRD in cattle. The *CXCR1* gene is a chemokine receptor that when stimulated by IL-8 (or CXCL8) attracts and activates neutrophils [11]. Interestingly, the increase in transcript abundance of *CXCR1* coincided with an increase in neutrophil count among the sick animals (Table 2), and its ligand IL-8 (CXCL8) also showed an increase in transcript abundance in these animals (P_nominal_ = 0.008, Additional file 1).

**Table 3:** Cytokine genes and receptors associated with bovine respiratory disease in calves

Gene	Effect	SE	F	Nominal P	P_corrected_	Time point
CCL16	5.15	0.727	50.14	0.00010	0.0087	Diagnosis
CXCR1	8.71	1.706	26.09	0.00092	0.077	Diagnosis
CCR1	4.56	0.901	25.65	0.00097	0.081	Diagnosis

Naïve lymphocytes that respond to infection in the lung express *CCR* genes 1–10 [12]. Specifically, human monocytes have been shown to respond to respiratory syncytial virus by increasing expression of CCR1 receptors [13]. Mice lacking the CCR1 gene produce little if any inflammatory responses during the acute phase of viral pneumonia [14]. The pneumonia virus of mice (PVM) produces similar chemokine responses as human respiratory syncytial virus (RSV). In this study, we detected an increase in the transcript abundance of *CCR1* at the time of illness. Moreover, of the five sick animals, one tested positive for bovine BRSV and two were positive for BCV.

The expression of *CCL16* has been widely reported in the liver [15], but its expression can also be induced in activated monocytes by IL-10, IFN-γ and bacterial lipopolysaccharide [16]. The cattle with BRD in this study displayed increased levels of *CCL16*. We detected a positive animal correlation between monocytes counts and CCL16; thus, a higher level of *CCL16* expression among animals with BRD could suggest that these animals have greater numbers of activated circulating monocytes.

Many of the previous studies of hematology parameters for calves available in the literature are limited by small sampling sizes and include only one breed or a specific cross of cattle [17–19]. A recent study by Leach et al. [1] is the only example of CBC data on a large population of 2182 crossbred calves. Our study sampled 796 crossbred calves at 120 days and 791 at 150 days for normal hematology parameters, and also includes hematology and cytokine gene expression data from animals that developed BRD (up to 192 days of age). In addition, the data presented here includes crossbred heifers and steers representing 18 breeds of origin.

### Summary

Complete blood counts can aid in the diagnosis of disease; thus, current information regarding the ranges of these values in beef cattle breeds may be useful veterinary tools. Our aim was to determine whether these values might provide insight into an animal’s subsequent health status. We have identified CBC values that were different in this set of animals between those that became sick and those that remained healthy at various time points. In addition, we identified differences among several cytokine genes and receptors that may also be useful biomarkers of BRD; however, it is critical that this study be replicated with a larger population of animals.

## Limitations

While we present a larger number of normal, healthy control animals for CBC, we were limited by the small number of cattle that were diagnosed with bovine respiratory disease. The cost to collect blood from all animals into Tempus RNA tubes was prohibitive for all 800 calves, and of the 200 animals sub-sampled for RNA, only 5 were diagnosed with illness. In general, illness was low for the season with only 13 of the 791 weaned calves diagnosed with BRD. Because BRD is a phenotypic measurement of symptoms, it is possible that some cases were not identified. We intend to repeat this experiment to increase the population size of sick animals and will use RNA-Seq to evaluate the sick and control animals from this study and the repeated study. This will allow us to detect the expression levels of all genes expressed in the leukocytes, including the genes evaluated in this study.

## Additional file

**Additional file 1.** Cytokine genes and receptors associated with bovine respiratory disease at the time of diagnosis in calves (N = 5 with BRD, N = 9 healthy control animals).

## Abbreviations

BCV: bovine coronavirus
BHV-1: bovine herpesvirus-1
BRD: bovine respiratory disease
BRSV: bovine respiratory syncytial virus
BVDV: bovine viral diarrhea virus
CBC: complete blood count
cDNA: complimentary deoxyribonucleic acid
EDTA: ethylenediaminetetraacetic acid
PCR: polymerase chain reaction
PVM: pneumonia virus of mice
RNA: ribonucleic acid
RNA-Seq: ribonucleic acid sequencing
RSV: respiratory syncytial virus
WBC: white blood cells
